# Phylogenetically diverse group of native bacterial symbionts isolated from root nodules of groundnut (*Arachis hypogaea* L.) in South Africa

**DOI:** 10.1016/j.syapm.2017.02.002

**Published:** 2017-06

**Authors:** Sanjay K. Jaiswal, Levini A. Msimbira, Felix D. Dakora

**Affiliations:** a Department of Chemistry, Tshwane University of Technology, Pretoria, South Africa; b Department of Crop Sciences, Tshwane University of Technology, Pretoria, South Africa

**Keywords:** IGS, RFLP, Phylogenetic, *Bradyrhizobium*, *Rhizobium*, Promiscuity, Nodulation

## Abstract

Groundnut is an economically important N_​2_-fixing legume that can contribute about 100–190 kg N ha^−1^ to cropping systems. In this study, groundnut-nodulating native rhizobia in South African soils were isolated from root nodules. Genetic analysis of isolates was done using restriction fragment length polymorphism (RFLP)-PCR of the intergenic spacer (IGS) region of 16S-23S rDNA. A total of 26 IGS types were detected with band sizes ranging from 471 to 1415 bp. The rhizobial isolates were grouped into five main clusters with Jaccard's similarity coefficient of 0.00–1.00, and 35 restriction types in a UPGMA dendrogram. Partial sequence analysis of the 16S rDNA, IGS of 16S rDNA-23S rDNA, *atpD*, *gyrB*, *gltA*, *glnII* and symbiotic *nifH* and *nodC* genes obtained for representative isolates of each RFLP-cluster showed that these native groundnut-nodulating rhizobia were phylogenetically diverse, thus confirming the extent of promiscuity of this legume. Concatenated gene sequence analysis showed that most isolates did not align with known type strains, and may represent new species from South Africa. This underscored the high genetic variability associated with groundnut *Rhizobium* and *Bradyrhizobium* in South African soils, and the possible presence of a reservoir of novel groundnut-nodulating *Bradyrhizobium* and *Rhizobium* in the country.

## Introduction

The use and cultivation of groundnut (*Arachis hypogaea* L.) dates back to 350 BC in its native South America where it has been used for thousands of years [1]. Traders were responsible for spreading groundnut from South America to Asia and Africa [18]. The Spanish and Portuguese explorers brought groundnut on their voyages to Africa. For example, West Africa Portuguese traders in the 16th century introduced the crop. Groundnut then flourished in many African countries and was incorporated into local traditional food cultures and was revered as a sacred food.

Nitrogen fixation by legume- rhizobia symbiosis plays a major role in sustaining soil health for crop production. However, this process is influenced by many factors, which include geographic location, soil type and host-plant genotypes, as well as the rhizobial symbiont itself [36]. Groundnut is reported to derive about 70–90% of its N requirements from symbiosis and to contribute an estimated amount of 100–190 kg N ha^−1^ to the cropping system [34]. In Africa, groundnut can obtain approximately 33–67% of its N nutrition from fixation [30] and fix up to 101 kg N ha^−1^ per cropping season [8].

Groundnut is generally nodulated by slow-growing rhizobia of the genus *Bradyrhizobium*, although effective fast-growing strains have also been reported to nodulate this legume [19]. *Bradyrhizobium* is a cosmopolitan and diverse group of microsymbionts capable of nodulating a variety of legumes, as well as the non-legume *Parasponia* [23]. Species of bradyrhizobia, such as *Bradyrhizobium japonicum*, *Bradyrhizobium elkanii*, *Bradyrhizobium lablabi*, *Bradyrhizobium yuanmingense* and *Bradyrhizobium iriomotense* are known to nodulate groundnut [51]. Additionally, fast-growing species of the genus *Rhizobium* can also nodulate groundnut, and they include *Rhizobium giardinii* and *Rhizobium tropici* [45].

The diversity of groundnut-nodulating rhizobia has been widely investigated using molecular techniques, which include the use of PCR-based methods to characterize the genetic relationships between rhizobial species.

Rhizobia are taxonomically diverse [55], and therefore require the use of well-tested, easy, quick techniques to differentiate microsymbionts at the genus, species and even strain level [15]. Restriction fragment length polymorphism (RFLP) analysis of 16S rRNA amplification using the polymerase chain reaction (PCR) provides a simplified method for characterization of rhizobial isolates at the molecular level [24], [32]. However, the use of the 16S rRNA gene alone as a phylogenetic marker in differentiating closely related species and strains within species has run into difficulties because of: (i) its presence as multiple copies in the genome of some bacteria, (ii) its susceptibility to genetic recombination and horizontal gene transfer, and (iii) its low divergence between closely related species [3], [13], [28], [46]. Thus, the IGS and housekeeping genes are currently in use as markers for molecular systematics and for estimation of the phylogenetic relationships among rhizobia. The sequences of 16S-23S rRNA give more coherent results, which are similar to DNA-DNA hybridization rather than 16S rRNA sequence analysis [54].

Based on the 16S-23S rRNA analysis, a higher level of diversity and heterogeneity was observed in groundnut bradyrhizobia in Canada [40], [48], China [57], [59], Cameroon [33], Argentina [34], [45], and other geographic regions [49]. However, despite these studies, the degree of genetic diversity among groundnut-nodulating rhizobia is still not properly understood. Previous studies using only five South African groundnut isolates showed some measure of diversity [41]. Therefore, the aim of this investigation was to obtain a complete understanding of the diversity present in groundnut-nodulating rhizobia in South African soils. To do this, firstly, a wide range of isolates was obtained from groundnut nodules in South Africa and they were analysed using IGS PCR-RFLP. Secondly, the gene sequences were determined for the 16S rDNA, IGS, *gln*II, *gyr*B, *glt*A and *atp*D genes located in the core genome, as well as the symbiotic genes *nifH* and *nodC* from selected isolates, and phylogenetic analysis of these genes was used to identify the bacteria.

## Materials and methods

### Rhizobial isolation and culture conditions

Root nodules were collected from groundnut plants grown at Klipladrift (26° 56′ 15.58″ S 29° 52′ 29.60″ E) in Mpumalanga Province, and Kwamhlanga (25° 25′ 48.04″ S 28° 42′ 43.85″ E) in Gauteng Province, South Africa. Sampling sites were chosen because groundnut was being introduced into these locations. None of the regions included in this study had any history of inoculation with rhizobial strains. Nodules were collected at 50% flowering of the groundnut crop. Nodules were surface-disinfected, squashed, and the nodule macerate was used to streak plates of yeast mannitol agar (YMA) medium, as described by Somasegaran and Hoben [40]. Pure single-colonies of the bacterial isolates were streaked on YMA agar containing 0.3% CaCO_3_ in McCartney bottles and preserved at 4 °C for later use.

### Nodulation assay

Healthy groundnut seeds were surface-sterilized by treatment with 70% ethyl alcohol for 1.5 min, washed with 3.5% NaOCl for 2 min, and then thoroughly rinsed with sterile distilled water five times. The surface-sterilized seeds were sown in sterile plastic pots containing sterilized sand, and they were watered twice a week with N-free plant nutrient solution [6]. After germination, the groundnut seedlings were thinned to one seedling per pot, and inoculated with 2 mL of a bacterial culture in the log phase (≈10^7^–10^8^ bacterial cells mL^−1^). Three replicate pots were used per isolate and three pots of uninoculated seedlings that received 2 mL sterile distilled water served as controls. After six weeks, the plants were harvested and visually examined for nodulation.

### Isolation of rhizobial DNA and PCR amplification of the IGS (16S-23S rDNA) region

Nodule bacterial genomic DNA was extracted using the GenElute™ Bacterial DNA Isolation Kit (Sigma-Aldrich, USA), according to the manufacturer’s instructions. The integrity of isolated DNA was checked on 1% agarose gel stained with ethidium bromide. The polymerase chain reaction (PCR) was carried out with 60–80 ng DNA in a 25 μL reaction volume containing 5× My Taq PCR buffer, 0.5 U Taq polymerase (Bioline, USA), and 10 pM each of the primers for the IGS region using a standard temperature profile ([2], [25], [27], [29], [35], [42], [43], [52]) in a thermal cycler (T100, Bio-Rad, USA). The amplified product (band) size was estimated from horizontal gel electrophoresis on 2% agarose gel stained with ethidium bromide using a standard DNA marker (GeneDirex, 1 kbp), and photographed using a gel documentation system (Geldoc™ XR+, Bio-Rad, USA).

### Restriction fragment length polymorphism (RFLP) of the IGS region

The PCR-amplified IGS region was digested with fast digest restriction endonucleases (*Hae*II and *Hind*III), following the manufacturer’s instructions (Thermo Scientific, Lithuania). The digested fragments were separated by horizontal gel electrophoresis on 3% agarose gel containing ethidium bromide. Electrophoresis was performed in tris-acetic acid EDTA (1X TAE) buffer at 85 V for 2.5 h and subsequently photographed under UV light with the Bio-Rad Gel documentation system.

### RFLP cluster analysis of the IGS regions

Only distinct, well-resolved, and unambiguous bands were scored, and faint bands were discarded. Bands ≤50 bp in size were not included for cluster analysis. The restriction enzyme-digested fragments were scored as: (1) in the presence of, and (0) in the absence of homologous bands. Thereafter, the similarity of strains tested was evaluated by a simple matching Jaccard similarity coefficient with the help of NTSYSpc 2.1 software [37], and a dendrogram was constructed from the distance matrix using the unweighted pair group method with arithmetic mean algorithm (UPGMA).

### PCR amplification, sequencing and phylogenetic analysis of 16S rDNA, IGS, housekeeping (*atpD*, *glnII*, *gyrB* and *gltA*) and symbiotic (*nodC* and *nifH*) genes

PCR amplification of 16S rDNA, *atpD*, *glnII*, *gyrB*, *gltA* and the symbiotic *nodC* and *nifH* genes of the rhizobial genome was carried out as described above for IGS-PCR amplification. The primers and thermal cycle conditions used are listed in [2], [25], [27], [29], [35], [42], [43], [52]. The PCR-amplified products of IGS, 16S rDNA, *atpD*, *glnII*, *gyrB*, *gltA* and symbiotic *nodC* and *nifH* were purified by the FavorPrep™ PCR Purification Kit (FAVORGEN, Sigma, USA). The purified samples were sequenced (Macrogen, Netherlands), and the quality of all sequences was checked using BioEdit 7.0.0 software [17]. NCBI GenBank databases were used to identify species closely related to the test isolates using the BLASTn program. The sequences were deposited in the GenBank database in order to obtain accession numbers after confirmation of the 3′ and 5′ direction (Table S2). Reference type sequences were selected in order to align the sequences of the test isolates using MUSCLE [9] for the construction of a phylogenetic tree created with the MEGA 6.0 program [44]. Phylogenetic trees were generated by Kimura’s 2-parameter model and the neighbor-joining algorithm [22], [38] with 1000 bootstrap support [12]. Nucleotide information was obtained from conserved, variable, parsimony-informative, and singleton regions using consensus sequences.

## Results

A total of 71 bacterial isolates were obtained from the root nodules of groundnut planted in South African soils, and 46 of the isolates elicited nodulation in groundnut (the homologous host) under glasshouse conditions. These authenticated rhizobial isolates were then genetically analyzed using various molecular tools.

### IGS PCR amplification

The IGS PCR-amplified product yielded polymorphic bands in the rhizobial isolates tested from groundnut. All the isolates revealed the presence of single bands, except TUTAHSA158 that produced more than one band in the 2% agarose gel. The IGS band lengths across the bacterial population varied from 471 to 1415 bp (Fig. S1A). The polymorphic bands obtained in this study successfully distributed the test rhizobial isolates into 24 groups, denoted by Roman numerals as IGS types I to XXIV (Table 1). IGS type X had the largest number (7) of isolates among the different polymorphic bands (Table 1).

**Table 1:** IGS type and restriction pattern of the PCR-amplified IGS (16S-23S rDNA) region of groundnut nodulating rhizobial strains.

Strains	Site of origin	Size (bp)	Restriction pattern type		
			IGS type	*Hind*III	*Hae*II
TUTAHSA10	Klipladrift	1415	I	E	O
TUTAHSA41	Klipladrift	1261	II	I	C
TUTAHSA45	Klipladrift	1261	II	J	M
TUTAHSA114	Kwamhlanga	1250	III	C	I
TUTAHSA116	Kwamhlanga	1250	III	L	I
TUTAHSA80	Klipladrift	1225	IV	E	I
TUTAHSA87	Klipladrift	1200	V	I	C
TUTAHSA31	Klipladrift	1060	VI	D	B
TUTAHSA84	Klipladrift	1041	VII	B	J
TUTAHSA19	Klipladrift	1039	VIII	G	B
TUTAHSA40	Klipladrift	1039	VIII	H	B
TUTAHSA51	Klipladrift	1039	VIII	H	N
TUTAHSA156	Kwamhlanga	1020	XVII	N	G
TUTAHSA157	Kwamhlanga	1200	V	G	P
TUTAHSA27	Klipladrift	980	IX	N	B
TUTAHSA7	Klipladrift	960	X	B	B
TUTAHSA155	Kwamhlanga	960	X	P	E
TUTAHSA61	Klipladrift	960	X	H	H
TUTAHSA75	Klipladrift	960	X	B	A
TUTAHSA97	Klipladrift	960	X	A	A
TUTAHSA159	Kwamhlanga	960	X	F	E
TUTAHSA160	Kwamhlanga	960	X	F	E
TUTAHSA158	Kwamhlanga	942	XI	–	E
TUTAHSA67	Klipladrift	936	XII	B	A
TUTAHSA73	Klipladrift	915	XIII	B	A
TUTAHSA154	Kwamhlanga	895	XIV	D	A
TUTAHSA115	Kwamhlanga	876	XV	A	H
TUTAHSA4	Klipladrift	866	XVI	K	H
TUTAHSA151	Kwamhlanga	856	XVII	A	A
TUTAHSA153	Kwamhlanga	856	XVII	A	G
TUTAHSA140	Kwamhlanga	838	XVIII	A	C
TUTAHSA143	Kwamhlanga	838	XVIII	F	L
TUTAHSA144	Kwamhlanga	819	XIX	A	F
TUTAHSA145	Kwamhlanga	819	XIX	A	F
TUTAHSA147	Kwamhlanga	819	XIX	D	F
TUTAHSA148	Kwamhlanga	819	XIX	A	A
TUTAHSA150	Kwamhlanga	819	XIX	D	A
TUTAHSA58	Klipladrift	819	XIX	K	D
TUTAHSA20	Klipladrift	781	XX	C	D
TUTAHSA118	Kwamhlanga	750	XXI	M	D
TUTAHSA17	Klipladrift	612	XXII	C	K
TUTAHSA152	Kwamhlanga	500	XXIII	C	A
TUTAHSA126	Kwamhlanga	471	XXIV	O	L

### IGS PCR-RFLP

**Fig. 1:**
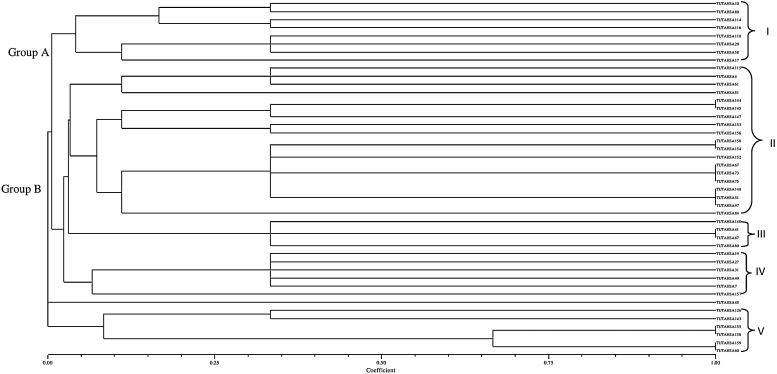
Dendrogram generated from *Hind*III and *Hae*II restriction enzyme digested IGS (16S-23 rDNA) RFLP restriction banding pattern of groundnut nodulating rhizobial isolates.

A much greater variation was found among the tested isolates using PCR-RFLP analysis of the 16S-23S rRNA intergenic spacer regions (Fig. S1B). IGS PCR-amplified products were digested with two 6-base cutting restriction endonucleases (*Hind*II and *Hae*II), and they revealed the presence of 35 IGS PCR-RFLP patterns (Fig. 1). The number of bands generated on 3% agarose gel stained with ethidium bromide ranged from 1 to 5 for *Hind*III, and 1 to 4 for *Hae*II (Fig. S1B). Test restriction enzymes *Hind*III and *Hae*II yielded same 15 (A-P) restriction banding pattern types. The *Hind*III restriction type A contained the highest number (8) of bacterial isolates, while *Hae*II restriction type A had nine isolates (Table 1). As a result of this analysis, a dendrogram was generated from the combined restriction profiles of *Hind*III and *Hae*II endonucleases using a binary matrix scoring 0/1 (0 in the absence and 1 in the presence of the restriction type), and by depicted similarity in the IGS region of the isolates (Fig. 1). From the IGS PCR-RFLP analysis, and subsequent UPGMA clustering, all isolates were grouped into five main clusters at Jaccard similarity coefficients of 0.00–1.00 (Fig. 1). Isolated strains were joined at the final Jaccard similarity coefficient level of 0.0. Cluster II contained the largest number (15) of isolates, while cluster III had the lowest number (4) of isolates (Fig. 1). Strain TUTAHSA45 was highly diverse compared to all the other tested isolates, since it stood independently (Fig. 1). Clusters I, II, III and IV formed major Group A joined together at a 0.01 similarity coefficient, while Cluster V and isolate TUTAHSA45 formed major Group B, which was highly diverse. Cluster I contained two sub-clusters joined at a similarity coefficient of 0.07, while Cluster II had three sub-clusters that joined together at a 0.03 similarity coefficient.

### Sequencing and phylogenetic analysis of the 16S rDNA region

For direct sequencing of 16S rDNA and IGS gene-amplified products, representative isolates from each cluster were randomly selected based on restriction fragment length polymorphism. The BLASTn analysis showed that the test isolates grouped with *Bradyrhizobium* and *Rhizobium* species groups.

### *Bradyrhizobium* group

**Fig. 2:**
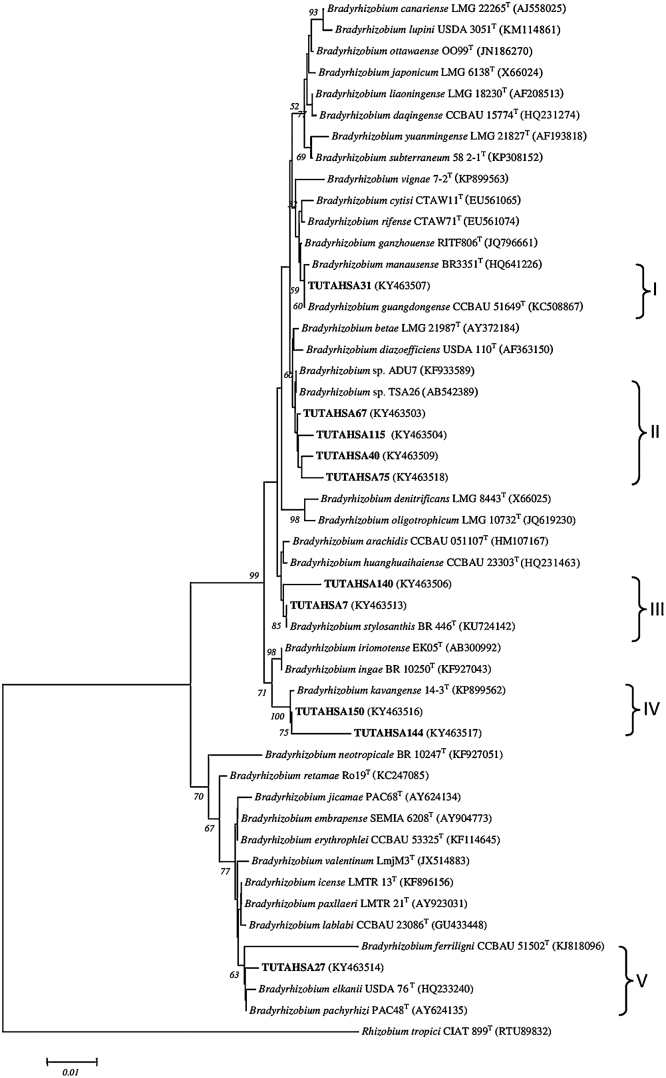

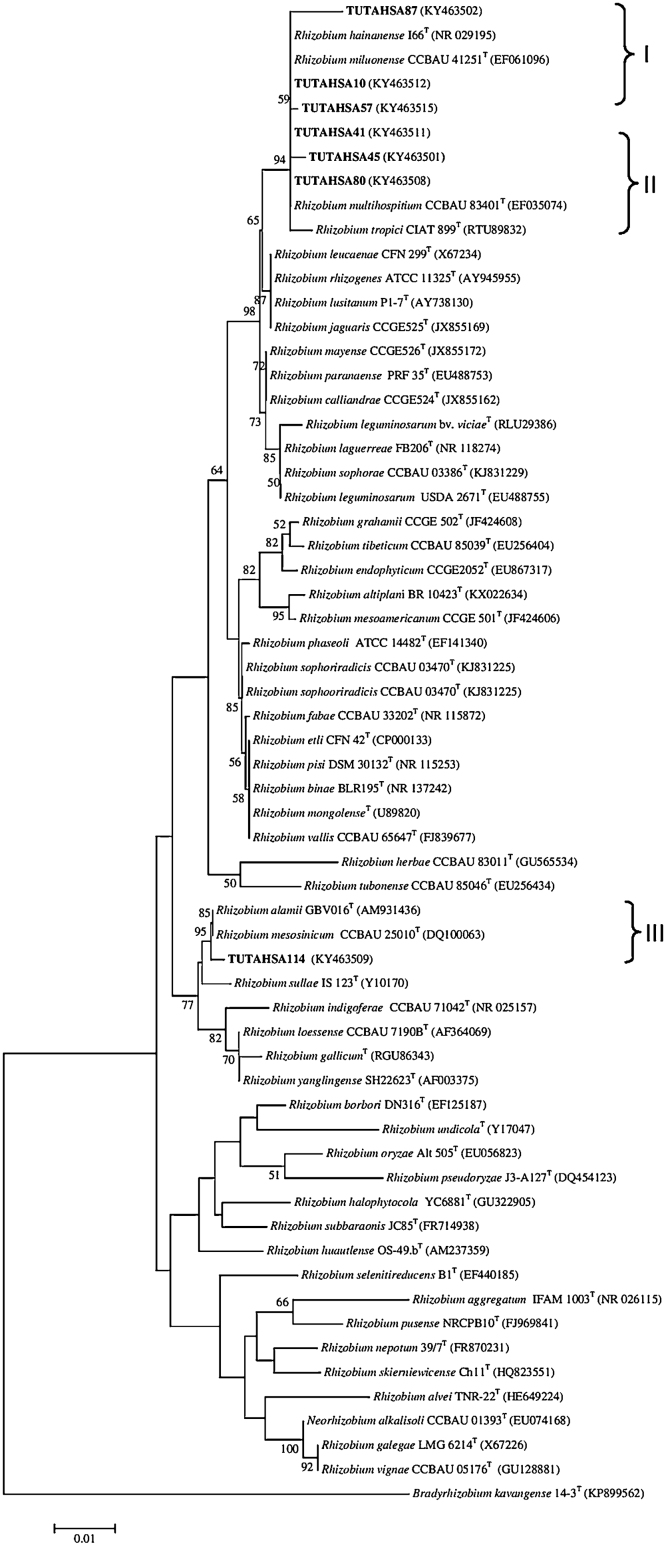
(a) The neighbour-joining phylogenetic relationships of groundnut nodulating *Bradyrhizobium* based on 16S rDNA sequence analysis. Groundnut nodulating microsymbiont are shown in bold with their nucleotide sequence accession numbers indicated in brackets. The significance of each branch is indicated by a bootstrap value = >50 are indicated for each node (1000 replicates). The scale bar represents the number of changes per nucleotide position. Phylogenetic analyses were conducted in MEGA6. (b) The neighbour-joining phylogenetic relationships of groundnut nodulating *Rhizobium* based on 16S rDNA sequence analysis. Groundnut nodulating microsymbiont are shown in bold with their nucleotide sequence accession numbers indicated in brackets. The significance of each branch is indicated by a bootstrap value = >50 are indicated for each node (1000 replicates). The scale bar represents the number of changes per nucleotide position.

Aligned 1110 bp sequences of 16S rDNA contained 911 conserved, 194 variable, 71 parsimony-informative and 123 singleton sites (Table S3). The phylogenetic tree constructed from the 16S rDNA sequences placed all isolates into five groups (Groups I–V) (Fig. 2a). Group I was formed by isolate TUTAHSA31 and type strains *Bradyrhizobium manausense* BR3352^T^ and *Bradyrhizobium guangdongense* CCBAU 51649^T^ with 60 bootstrap support. Isolates TUTAHSA67, TUTAHSA40, TUTAHSA115 and TUTAHSA75 showed their close relationship with *Bradyrhizobium* sp. ADU7 isolated from groundnut in China with 99.4–99.9% sequence identity in Group II. In Group III, isolates TUTAHSA140 and TUTAHSA7 clustered with *Bradyrhizobium stylosanthis* BR 446^T^ with 99.1–100% sequence identity. Isolates TUTAHSA150 and TUTAHSA144 showed a close relationship with *Bradyrhizobium kavangense* 14-3^T^ with high 100 bootstrap support in Group IV. In Group V, isolate TUTAHSA27 was aligned with *B. elkanii* USDA76^T^, *Bradyrhizobium pachyrhizi* PAC 48^T^ and *Bradyrhizobium ferriligni* CCBAU 51502^T^ with 63 bootstrap support.

### *Rhizobium* group

In the *Rhizobium* group, the 825 aligned nucleotide sequences had 670 conserved, 155 variable, 89 parsimony-informative and 66 singleton sites (Table S3). In the phylogenetic tree, isolates TUTAHSA87, TUTAHSA10, TUTAHSA57, TUTAHSA41, TUTAHSA45 and TUTAHSA80 grouped with *R. tropici*-related type strains (*Rhizobium hainanense* I66^T^, *Rhizobium miluonense* CCBAU 41251^T^ and *Rhizobium multihospitium* CCBAU 83401^T^) in Groups I and II with 94 bootstrap support. In Group III, isolate TUTAHSA114 was closely related to *Rhizobium alamii* and *Rhizobium mesosinicum* with 95 bootstrap value (Fig. 2b).

### Sequencing and phylogenetic analysis of the IGS region

Due to the divergence of the IGS region among *Bradyrhizobium* species, phylogenetic studies of this region were considered to be more appropriate. The sequences generated from the IGS region were used to align with type strain IGS sequences selected from GenBank. Based on partial IGS sequence comparisons with the GenBank references, some isolates were identified as *Bradyrhizobium* sp. and others as *Rhizobium* sp. The nucleotide sequence analysis results are indicated in Table S3.

**Fig. 3:**
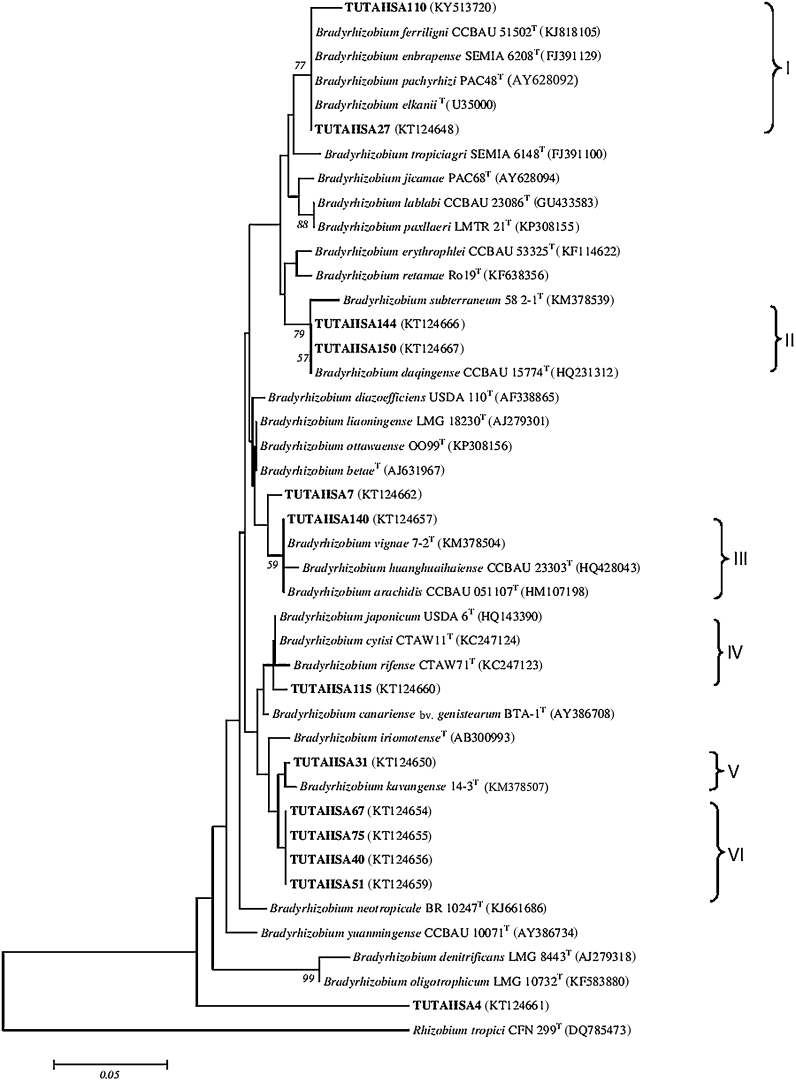

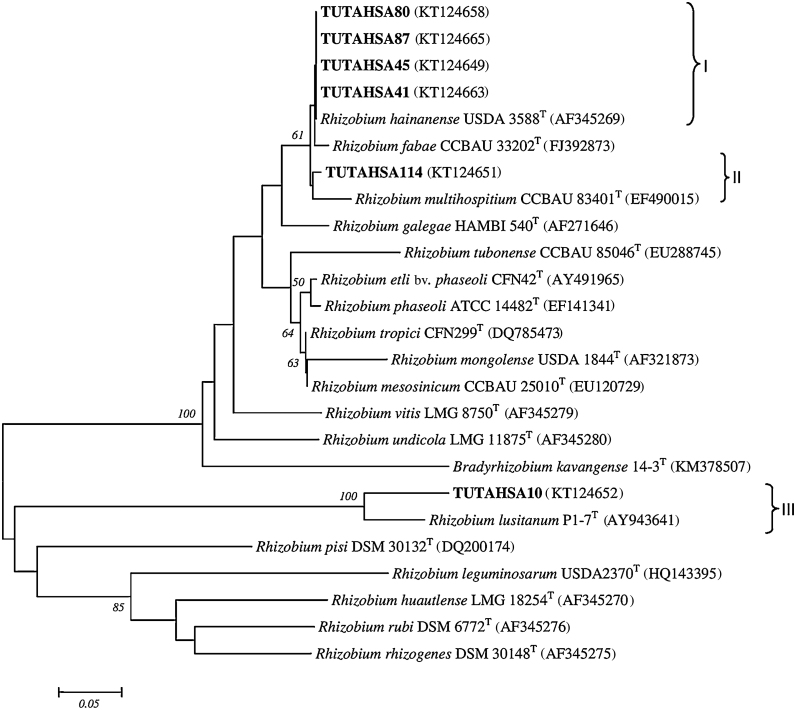
(a) The neighbour-joining phylogenetic relationships of groundnut nodulating *Bradyrhizobium* based on IGS (16S-23S rDNA) sequence analysis. Groundnut nodulating microsymbiont are shown in bold with their nucleotide sequence accession numbers indicated in brackets. The significance of each branch is indicated by a bootstrap value = >50 are indicated for each node (1000 replicates). The scale bar represents the number of changes per nucleotide position. Phylogenetic analyses were conducted in MEGA6. (b) The neighbour-joining phylogenetic relationships of groundnut nodulating *Rhizobium* based on IGS (16S-23S rDNA) sequence analysis. Groundnut nodulating microsymbiont are shown in bold with their nucleotide sequence accession numbers indicated in brackets. The significance of each branch is indicated by a bootstrap value = >50 are indicated for each node (1000 replicates). The scale bar represents the number of changes per nucleotide position. Phylogenetic analyses were conducted in MEGA6.

As for 16S rDNA, the two phylogenetic trees were constructed from the IGS sequences of test isolates with *Bradyrhizobium* and *Rhizobium* species groups (Fig. 3a and b).

### *Bradyrhizobium* group

The topology of the IGS phylogram was similar to the 16S rDNA phylogeny but with a slight variation in the isolate placements in the trees. The test isolates in the *Bradyrhizobium* tree were further divided into six (I–VI) distinct groups (Fig. 3a). In Group I, isolates TUTAHSA27 and TUTAHSA 110 clustered with *B. ferriligni*, *Bradyrhizobium embrapense*, *B. pachyrhizi* and *B. elkanii* with 77 bootstrap support and 99.6% sequence similarity. In Group II, isolates TUTAHSA144 and TUTAHSA150 were aligned with *Bradyrhizobium subterraneum* with 97.8–98.4% sequence similarity. In Group III, TUTAHSA140 aligned with *Bradyrhizobium huanghuaihaiense* with 59 bootstrap support, while TUTAHSA7 was an outgroup. Isolate TUTAHSA115 was proximally related to the type strains *B. japonicum*, *Bradyrhizobium cytisi* and *Bradyrhizobium rifense* in Group IV. Isolates TUTAHSA51, TUTAHSA67, TUTAHSA75, and TUTAHSA40 were however clustered together and stood alone in Group VI. Isolate TUTAHSA4 also stood alone but formed an outgroup in the phylogram.

### *Rhizobium* group

In the *Rhizobium* group, isolates TUTAHSA41, TUTAHSA87, TUTAHSA10, TUTAHSA114, TUTAHSA45 and TUTAHSA80 clustered with different *Rhizobium* species in three (I–III) distinct groups. Isolates TUTAHSA80, TUTAHSA87, TUTAHSA45 and TUTAHSA41 were closely related to strain *R. hainanense* in Group I, while isolate TUTAHSA114 formed a close relationship with *R. multihospitium* in Group II. Isolate TUTAHSA10 grouped with *Rhizobium lusitanum* PI-7^T^ with high (100) bootstrap support (Fig. 3b).

### Analysis of the housekeeping genes

For a clear resolution of the phylogenetic analysis, the four housekeeping genes *gyrB*, *atpD*, *glnII* and *gltA*, which are highly conserved among bacteria belonging to the *Rhizobiales* and encode DNA gyrase subunit B, ATP synthase beta chain, glutamine synthase II and citrate synthase, respectively, were selected for further studies.

Selected representative isolates from RFLP analysis yielded amplified bands of the four genes. Fewer sequences were used in *atpD* and *gltA* phylogeny due to difficulties in PCR amplification and/or bad sequence results. Thus, the phylogenetic analysis of these genes was performed individually (Figs. S2–S5). The sequences of *glnII*, *gyrB*, *atpD* and *gltA* were aligned with local and type strain nucleotide sequences obtained from GenBank. The length of the alignments used was 369 bp for *atpD*, 424 for *glnII*, 226 for *gltA* and 561 bp for *gyrB*. Of the four gene sequences, *gltA* was the shortest and the lowest informative with only 56 informative positions. The highest level (314) of parsimony-informative sites was observed in *gyrB* (Table S3). As in the 16S rDNA and IGS phylograms, *Bradyrhizobium* and *Rhizobium* groups were also observed in *glnII* and *gyrB* phylogenies. Since not all test isolates were included due to the problem of PCR amplification with the *atpD* gene, only the *Bradyrhizobium* group-aligned isolates were observed in the phylogeny. The phylogenetic tree constructed with test isolates and type strains of *Bradyrhizobium* and *Rhizobium* species for the four genes did not give consistent or the same topology results in all trees, except for isolate TUTAHSA27 that clustered with *B. elkanii* and *B. pachyrhizi*. In all trees, except for *glt*A, isolates TUAHSA67, TUTAHSA75 and TUTAHSA51 consistently clustered together and shared 99.4-100% sequence similarity with each other while forming a separate branch within the genus *Bradyrhizobium*. In the *glt*A phylogram, the test isolates showed some discordance when compared to the phylograms of the other test housekeeping genes. Isolates TUAHSA67, TUTAHSA75 and TUTAHSA51 were grouped with *Rhizobium* species in the *gltA* phylogram.

### Concatenated sequence analysis

**Fig. 4:**
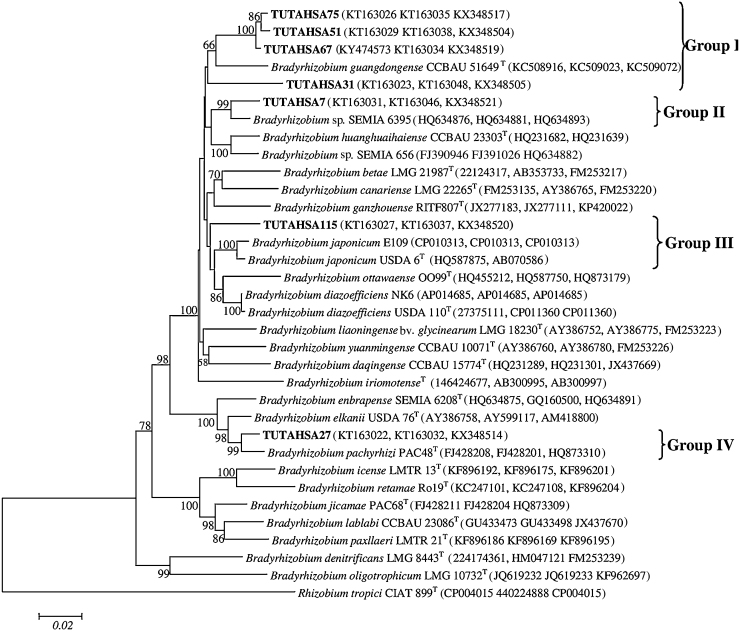
Phylogenetic relationships of groundnut nodulating *Bradyrhizobium* based on multilocus concatenated sequence analysis of the *atpD, glnII* and *gyrB* genes. The significance of each branch is indicated by a bootstrap value = >50 are indicated for each node (1000 replicates). The scale bar represents the number of changes per nucleotide position. Phylogenetic analyses were conducted in MEGA6.

Aligned sequences of *glnII*, *gyrB*, *gltA* and *atpD* were used to construct the concatenated phylogeny. Due to the unavailability of either PCR-amplified product or nucleotide sequences of *atpD* and *gltA* regions of some isolates, two (*atpD* + *glnII* + *gyrB* and *glnII* + *gyrB* + *gltA*) separate concatenated trees were constructed for *Bradyrhizobium* and *Rhizobium* species. The concatenated sequences of *atpD* + *glnII* + *gyrB* regions of *Bradyrhizobium* contained 1355 analyzed sites of which 824 were conserved, 525 were variable, 317 were parsimony-informative and 208 were singletons (Table S3). The tree built with these concatenated sequences resulted in four groups (Fig. 4). In the first group, isolates TUTAHSA67, TUTAHSA51 and TUTAHSA75 were proximally related to *B. guangdongense* with 95.2–95.4% sequence similarity and 66 bootstrap support. Isolate TUTAHSA115 showed a proximal relationship with *B. japonicum* and *Bradyrhizobium diazoefficiens* in Group III, while isolate TUTAHSA 31 stood alone as an outgroup without any type strains in Group I. Isolate TUTAHSA7 in Group II clustered with *Bradyrhizobium* sp. SEMIA 6395 isolated from the host *Calliandra houstoniana* in Brazil with high 99 bootstrap support. In Group IV, isolate TUTAHSA27 was closely related to *B. pachyrhizi* with high 99 bootstrap support and 98% sequence similarity.

**Fig. 5:**
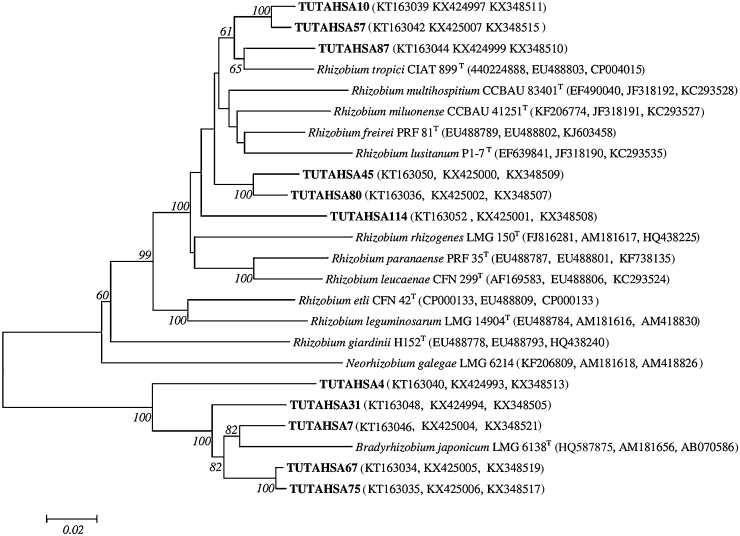
Phylogenetic relationships of groundnut nodulating *Rhizobium* based on multilocus concatenated sequence analysis of the *glnII*, *gltA* and *gyrB*, genes. The significance of each branch is indicated by a bootstrap value = >50 are indicated for each node (1000 replicates). The scale bar represents the number of changes per nucleotide position. Phylogenetic analyses were conducted in MEGA6.

The second phylogenetic tree of *glnII* + *gltA* *+* *gyrB* concatenated sequences gave a clear view of the test isolates related to *Rhizobium* (Fig. 5). The concatenated sequences of *glnII* + *gltA + gyrB* regions of *Rhizobium* contained 1211 analyzed sites of which 691 were conserved, 520 were variable, 409 were parsimony-informative and 111 were singletons (Table S3). Isolate TUTAHSA87 clustered with *R. tropici* with 65 bootstrap support and 94.5% sequence similarity. In this phylogram, most of the isolates stood alone without any type strains. For example, isolates TUTAHSA10 and TUTAHSA57 stood alone and were closely related with 98.2% sequence similarity. Even isolates TUTAHSA45 and TUTAHSA80 stood out, since they clustered together with 97.9% sequence similarity, whereas isolate TUTAHSA114 also stood alone but without any type strains. Isolates TUTAHSA4, TUTAHSA31, TUTAHSA7, TUTAHSS67 and TUTAHSA75 formed an outgroup with *B. japonicum*.

### Sequence and phylogenetic analysis of the *nifH* gene

**Fig. 6:**
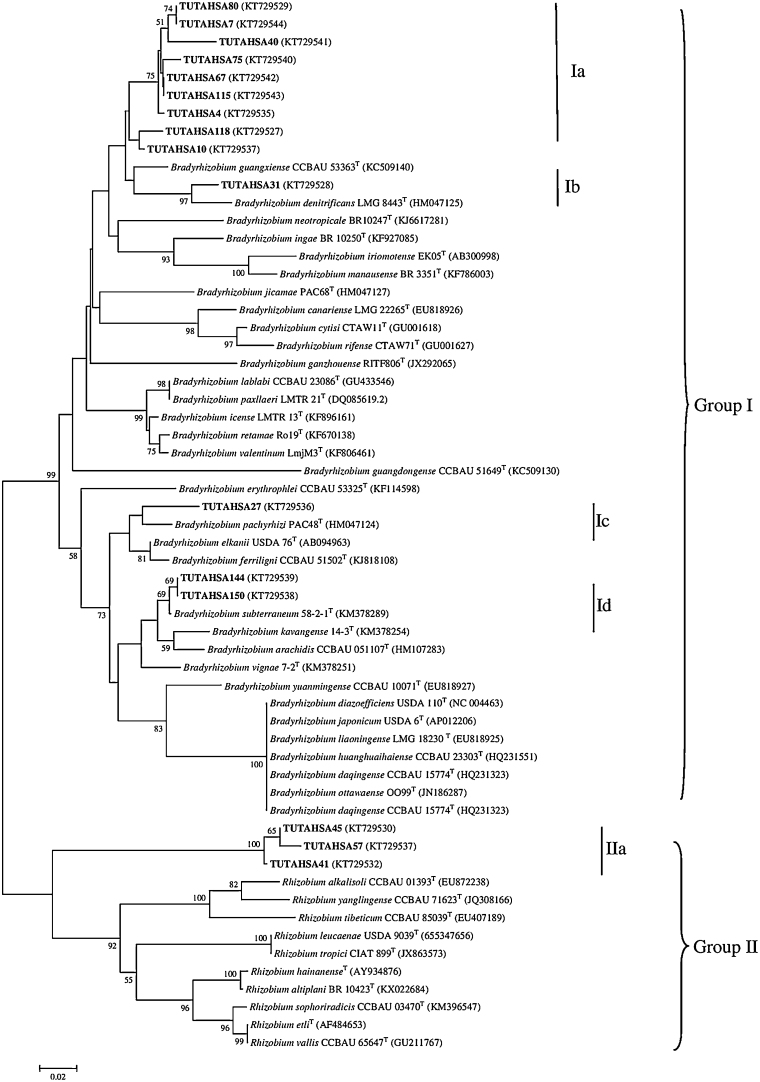
The neighbour-joining phylogenetic relationships of groundnut nodulating microsymbionts based on *nifH* sequence analysis. The significance of each branch is indicated by a bootstrap value = >50 are indicated for each node (1000 replicates). The scale bar represents the number of changes per nucleotide position.

A single band of approximately 800 bp was observed after PCR amplification of the *nifH* region of each isolate. The *nifH* sequences of test isolates were aligned with type sequences of *Bradyrhizobium* and *Rhizobium* strains and nucleotide sequence information is indicated in Table S3. In the *nifH* phylogenetic tree, most of the test isolates (TUTAHSA80, TUTAHSA7, TUTAHSA40, TUTAHSA75, TUTAHSA67, TUTAHSA115, TUTAHSA4, TUTAHSA118 and TUTAHSA10) formed a monophyletic group without any type strains in *Bradyrhizobium* Group I (Subgroup Ia) (Fig. 6). The closest type strain with these isolates was *Bradyrhizobium guangxiense* with 95.5–96% sequence identity. Isolates TUTAHSA31 and TUTAHSA27 showed a close relationship with *Bradyrhizobium denitrificans* and *B. pachyrhizi* in Subgroups Ib and Ic, respectively. In Subgroup Id, isolates TUTAHSA144 and TUTAHSA150 shared their *nifH* sequences with *B. subterraneum* with 69 bootstrap support and 96-99.5% sequence similarity. In *Rhizobium* Group II, a monophyletic group of isolates (TUTAHSA45, TUTAHSA57 and TUTAHSA41) was also observed as Subgroup IIa.

### PCR amplification of the *nodC* symbiotic region

The *nodC* symbiotic region of the isolates was amplified using different primer pairs ([2], [25], [27], [29], [35], [42], [43], [52]). All test bradyrhizobial isolates (TUTAHSA67, TUTAHSA115, TUTAHSA4, TUTAHSA75, TUTAHSA40, TUTAHSA80, TUTAHSA7, TUTAHSA118, TUTAHSA31, TUTAHSA27, TUTAHSA140, TUTAHSA144 and TUTAHSA150) identified by 16S rDNA, IGS and housekeeping genes yielded a single band of approximately 300 bp as a *nodC*-amplified product using nodCp8 and nodCIf primer pairs. A single band of approximately 1000 bp was obtained as a PCR-amplified product for the rhizobial isolates TUTAHSA114, TUTAHSA45, TUTAHSA80, TUTAHSA41 and TUTAHSA87 using nodCFn and nodCI primer pairs.

## Discussion

The N_2_-fixing ability of groundnut is essential for sustainable yields and economic returns, especially in less developed countries [11]. A common problem encountered by groundnut farmers and growers of other legumes is the presence of low symbiotically efficient native microsymbionts in the soil. Although legumes are generally nodulated by indigenous root-nodule bacteria [33], inoculation of groundnut with selected rhizobial strains has been shown to improve crop yields [5]. In this study, all the test rhizobial isolates were able to form effective nodules with groundnut as their homologous host under glasshouse conditions. The isolated groundnut-nodulating bacterial symbionts consisted of both fast-growers and slow-growers. Approximately 66% of the isolates grew on YMA plates after 6 days incubation, while the colonies of 34% of the isolates took 3 days to appear on YMA plates. These results are in contrast to those of Zhang et al. [59] and Saleena et al. [39] who detected only slow-growers as the sole occupants of groundnut root nodules. However, our results are consistent with those of Taurian et al. [45] who found both fast- and slow-growing, nodule-forming, N_2_-fixing bacteria in the root nodules of groundnut.

Knowledge of indigenous soil rhizobial populations is essential for selecting potential inoculant strains, as well as prior to the application of foreign inoculant strains [33]. Based on morphological, physiological and 16S rDNA-RFLP analysis of root nodules, a considerably large diversity has been found among rhizobia-nodulating groundnut in different countries [34], [45], [47], [58], [57]. In this study, the analysis of IGS-restriction fragment length polymorphism with two restriction endonucleases revealed the presence of 35 restriction types that showed huge diversity with 24 IGS types of groundnut-nodulating bacteria. This contrasts with the findings of Yang et al. [57], Ngo Nkot et al. [33] and Wang et al. [51], who respectively found only three, eight and two IGS types in their studies of groundnut rhizobia. Furthermore, our data also showed that all the test rhizobial isolates from South Africa had a very low (0.00) final similarity coefficient (Fig. 1) with a variable range (471–1415 bp) for the IGS PCR-amplified products. IGS length variability between and among rhizobial species has been previously reported [4], [21], [24]. The presence of different size bands can be explained by the variation in the conserved block within the IGS region [58], and the presence or absence of tRNA [16], [50], [53], [57].

The fragments produced in this study were smaller than those obtained by Yang et al. [57] and Yang and Zhou [58], and this could be attributed to the different primers used. In this study, some rhizobial isolates showed the same length in the IGS region but differed in restriction sites. However, it is known that the presence of polymorphic bands from the IGS region does not necessarily indicate differences in restriction sites [24]. Oddly, only strain TUTAHSA158 yielded more than one band from IGS amplification. This could possibly be due to the insertion of various tRNA genes in the IGS region [21], the heteroduplex DNA structure of single-stranded DNA [20], [24], or the existence of several copies of the rrs operon.

The IGS PCR-amplified products of some test rhizobial isolates (e.g. TUTAHSA4, TUTAHSA20, TUTAHSA41, TUTAHSA58, TUTAHSA87, TUTAHSA118, TUTAHSA17 and TUTAHSA126) could not be digested with the restriction enzymes *Hind*III and *Hae*II. This could have been due to the absence of restriction sites for the endonuclease enzymes used in the IGS sequences [33]. It was also found that cluster analysis of the RFLP data showed no linkage between strain clustering and the location from where groundnut nodules were collected for bacterial isolation. In fact, rhizobial isolates from the same origin could be seen in different clusters, while isolates from different origins were also found in the same group.

The observed variation in the IGS region of isolates in this study was consistent with the findings of Wang et al. [51] and Nievas et al. [34], who similarly found genetic variability among groundnut-nodulating rhizobia in China and Argentina. The results of the current study therefore showed that nodulation of groundnut as a host plant was elicited by an extremely diverse group of microsymbionts, and that two rhizobial genera induced nodulation of groundnut in South African soils.

Phylogenetic analysis of the selected microsymbionts using 16S rDNA, IGS, *atpD*, *gyrB*, *gltA* and *glnII* gene sequences revealed *Bradyrhizobium* and *Rhizobium* as the major predominant symbionts of groundnut. These data confirmed the high level symbiotic promiscuity of this legume. The fact that all the selected test isolates (both *Bradyrhizobium* and *Rhizobium*) could, in fulfilment of Koch’s postulates, form effective root nodules on groundnut (the homologous host) is in contrast to the results of Chen et al. [7] who found that only species of *Bradyrhizobium* nodulated groundnut, whereas Wong et al. [56] reported that groundnut nodules formed by fast-growing rhizobia were ineffective.

To test the robustness of the techniques used in the current study, two separate concatenated trees were constructed for *Bradyrhizobium* and *Rhizobium*. With three-gene (*atpD* *+* *glnII* + *gyrB*) concatenated tree analysis of *Bradyrhizobium*, the groundnut test isolates fell into four phylogenetic groups (Groups I–IV). Isolate TUTAHSA27 in Group IV shared high identity with *B. pachyrhizi* and *B. elkanii* with 97.1–98% sequence similarity, which interestingly was consistent in all individual housekeeping phylogenetic trees. All the test isolates in Group I could be defined as different new lineages. To date, eight defined *Bradyrhizobium* species (namely, *B. japonicum*, *B. elkanii*, *B. lablabi*, *B. yuanmingense*, *B. iriomotense*, *B. guangxiense*, *B. guangdongense* and *Bradyrhizobium arachidis*) and an unidentified *Bradyrhizobium* sp. have been reported to be capable of nodulating groundnut [7], [26], [31], [33], [39], [48], [49], [51], [59]. In Group I, isolates TUTAHSA67, TUTAHSA51 and TUTAHSA75 were closely related to *B. guangdongense* with 95.2–95.4% sequence identity. Isolate TUTAHSA115 was closely related to *B. japonicum* and *B. diazoefficiens* in the phylogram.

Furthermore, the new lineages were assessed in a second concatenated (*glnII* + *gyrB* + *gltA*) *Rhizobium* phylogram (Fig. 5), and all the groundnut-nodulating test isolates (namely, TUTAHSA87, TUTAHSA10, TUTAHSA57, TUTAHSA45, TUTAHSA80, and TUTAHSA114) grouped with *Rhizobium* species. This finding agreed with the results of Taurian et al. [45] and El-Akhal et al. [10] that groundnut rhizobia were phylogenetically related to *R. giardinii* and *R. tropici.*

However, the phylogenetic study of individual and concatenated genes revealed that many of these South African isolates were novel species, since they clustered with both *Bradyrhizobium* and *Rhizobium* species groups, and some were not even positioned in the tree. Thus, the taxonomic position of rhizobia nodulating groundnut is still not well defined. Therefore, isolates have been named by reference to the host plant as *Bradyrhizobium* sp. (*Arachis*) [10], [14], [45], [49].

In this study, the phylogeny of the *nifH* gene showed consistency with the core (housekeeping) gene phylogenies (Fig. 5). For example, isolates in Groups Ia and IIa formed a monophyletic group without any reference type strains in the *nifH* phylogeny, which was the same for the core genes. This suggested that they had the same evolutionary history for the chromosomal and symbiotic genes.

Considered together, the results of this study suggested that PCR-RFLP analysis of the 16S-23S rDNA IGS region in rhizobial isolates had sufficient discriminatory power to group chromosomally closely related strains based on the simple, reproducible results of restriction fragments. Combined data analysis from various restriction enzymes enabled the relatedness between 16S-23S rDNA IGS regions to be estimated. Phylogenetic analysis from this study revealed high-level promiscuity of groundnut, since it was nodulated by a diverse group of microsymbionts. The sequence alignment of the isolated strains with a divergent group of rhizobial strains further emphasized that groundnut was a highly promiscuous legume. The results showed the presence of abundant, widely distributed, diverse and novel types of native *Rhizobium* and *Bradyrhizobium* species in South African soils. Therefore, identifying indigenous rhizobial populations with high symbiotic efficiency could help increase groundnut yield and quality.
