# Supplementary material for: Phylogenetically diverse group of native bacterial symbionts isolated from root nodules of groundnut (Arachis hypogaea L.) in South Africa
Source: Syst Appl Microbiol. 2017 Jun;40(4):215–26. doi: 10.1016/j.syapm.2017.02.002 (PMC5460907; doi:10.1016/j.syapm.2017.02.002)
Supplement: Supplementary file 1 [file mmc1.pdf]

Phylogenetically diverse group of bacterial symbionts isolated from root nodules of groundnut (*Arachis hypogaea*) in South Africa

[1]Sanjay K Jaiswal, [2]Levini A Msimbira, [1]Felix D Dakora

[1]Department of chemistry, [2]Department of crop Sciences, Tshwane University of Technology, Pretoria, South Africa

Table S1

Primers and PCR temperature profiles used for DNA amplification.

Gene name	Primer Sequence (5' – 3')	Temperature profile	References
16S rDNA	fD1 (9-29)AGAGTTTGATCCTGGCTCAG<br>rD1 (1474-1494)CTTAAGGAGGTGATCCAGCC	5 min 95℃, 35 X (1 min 95 ℃, 1 min 55 ℃, 1 min 72℃), 10 min 72	[52]
IGS<br>(16S-23S rDNA)	FGPS1490-72 TGCGGCTGGATCACCTCCTT<br>FGPL132-38 CCGGGTTTCCCCATTCGG	5 min 95℃, 35 X (1 min 95 ℃, 1 min 55 ℃, 1 min 72℃), 10 min 72˚C	[2]
*glt*A	CSGCCTTCTAYCAYGACTC<br>GGGAGCCSAKCGCCTTCAG	5 min 94℃, 20 X [30 s 94℃, 30 s 63℃ (-0.5℃\cycle), 90s 72℃], 25 X (30 s 94℃, 30 s 54.5 ℃, 90s 72℃), 10 min 72˚C	[29]
*gln*ll	13f-AAGCTCGAGTACATCTGGCTCGACGG<br>681r-SGAGCCGTTCCAGTCGGTGGTGTCG	2 min 95℃, 35 X (45 s 95 ℃, 30 s 65 ℃, 90 s 72℃), 10 min 72˚C	[42]
*gyr*B	343f-TTCGACCAGAAYTCCTAYAAGG<br>1043r-AGCTTGTCCTTSGTCTGCG	10 min 95℃, 35 X (30 s 94℃, 30 s 58 ℃, 1 min 72℃), 10 min 72˚C	[27]
*atp*D	189f- TCTGGTCCGYGGCCAGGAAG<br>804r-CGACACTTCCGARCCSGCCTG	2 min 95℃, 35 X (45 s 95 ℃, 30 s 65 ℃, 90 s 72℃), 10 min 72˚C	[42]
*nif*H	28f-TACGGNAARGGSGGNATCGGCAA<br>809r-AGCATGTCYTCSAGYYTCNTCCA	5 min 94℃, 20 X [30 s 94℃, 30 s 65℃ (-0.5˚C / cycle), 90 s 72℃], 25 X (30 s 94℃, 30 s 65℃, 90 s 72℃), 10 min 72˚C	[35]
nodC1<br>*nodCp8*	5'GTC GAT TGC MRG TCA AGA CTA CG 3'<br>5'GCC AGG TCT IGT TGC GAT TGC TC 3'	30 s 94℃, 40 X [30s 94℃, 1 min 57℃, 30s 72℃], 5 min 72℃	[43]
$nodCf_n$<br>*nodCI*	5'AGGTGGTYGAYGACGGTTC3'<br>5'CGYGACAGCCANTCKCTATTG3'	30 s 94℃, 40 X [30s 94℃, 1 min 55.4℃, 30s 72℃], 5 min 72℃	[25]

Table S2

Accession numbers of the gene sequences of the soybean nodulating isolates used for the phylogenetic study

Strain	IGS	*16S rDNA*	*atpD*	*glnII*	*gyrB*	*gltA*	*nifH*
TUTAHSA27	KT124648	KY463514	KT163022	KT163032	KX348514		KT729536
TUTAHSA45	KT124649	KY463501		KT163050	KX348509	KX425000	KT729530
TUTAHSA31	KT124650	KY463507	KT163023	KT163048	KX348505	KX424994	KT729528
TUTAHSA114	KT124651	KY463509		KT163052	KX348508	KX425001	
TUTAHSA10	KT124652	KY463512		KT163039	KX348511	KX424997	KT729533
TUTAHSA67	KT124654	KY463503	KY474573	KT163034	KX348519	KX425005	KT729542
TUTAHSA75	KT124655	KY463518	KT163026	KT163035	KX348517	KX425006	KT729540
TUTAHSA40	KT124656	KY463509	KT163030		KX348518		KT729541
TUTAHSA140	KT124657	KY463506	KT163028		KX348522	KX425003	
TUTAHSA80	KT124658	KY463508		KT163036	KX348507	KX425002	KT729529
TUTAHSA51	KT124659		KT163029	KT163038	KX348504		
TUTAHSA115	KT124660	KY463504	KT163027	KT163037	KX348520		KT729543
TUTAHSA4	KT124661			KT163040	KX348513	KX424993	KT729535
TUTAHSA7	KT124662	KY463513	KT163031	KT163046	KX348521	KX425004	KT729544
TUTAHSA41	KT124663	KY463511		KT163049		KX424998	KT729532
TUTAHSA57	KT124664	KY463515		KT163042	KX348515	KX425007	KT729537
TUTAHSA87	KT124665	KY463502		KT163044	KX348510	KX424999	
TUTAHSA144	KT124666	KY463517	KT163024	KT163051			KT729539
TUTAHSA150	KT124667	KY463516			KX348516		KT729538
TUTAHSA17				KT163041			
TUTAHSA118				KT163045		KX424995	KT729527
TUTAHSA126				KT163047			KT428748
TUTAHSA110	KY513720			KT163053			

Table S3

Information of nucleotide sequences used for phylogenetic analysis

Locus	No. of strains used for tree construction	Nucleotide sequence information				Total*	Frequency T/C/A/G (%)
		Conserved (C)	Variables (V)	Parsimony-informative (Pi)	Singleton (S)		
16S rDNA *Bradyrhizobium*	49	911(82.1)	194(17.5)	71(6.4)	123(11.1)	1110	20.4/23.5/24.8/31.2
16S rDNA *Rhizobium*	62	670 (81.2)	155 (18.8)	89 (10.8)	66 (8.0)	825	20.4/21.7/26.1/31.7
ITS *Bradyrhizobium*	43	397(33.0)	759(63.0)	401(33.3)	353(29.3)	1204	24.6/26.5/17.0/31.9
ITS *Rhizobium*	25	212 (18.8)	864 (76.7)	633 (56.2)	216 (19.2)	1127	24.8/23.9/20.7/30.6
atpD	45	242 (65.6)	127 (34.4)	73 (19.8)	54 (14.6)	369	19.9/31.1/15.7/33.3
glnII	97	233 (54.9)	191 (45.0)	163 (38.4)	28 (6.6)	424	20.2/29.8/16.8/33.2
gltA	33	146 (64.6)	80 (35.4)	56 (24.8)	24 (10.6)	226	21.7/36.1/15.3/27.0
gyrB	77	209 (37.3)	352 (62.7)	314 (56.0)	38 (6.7)	561	21.7/30.0/16.8/31.5
atpD+glnII+ gyrB	34	824 (60.8)	525 (38.7)	317 (23.4)	208 (15.4)	1355	20.4/30.8/15.9/32.9
glnII +gltA+ gyrB	24	691 (57.1)	520 (43.0)	409 (33.8)	111 (9.1)	1211	21.6/30.5/16.7/31.1
nifH	57	111 (53.4)	97 (46.6)	89 (43.2)	08(3.4)	209	18.5/28.4/20.5/32.6

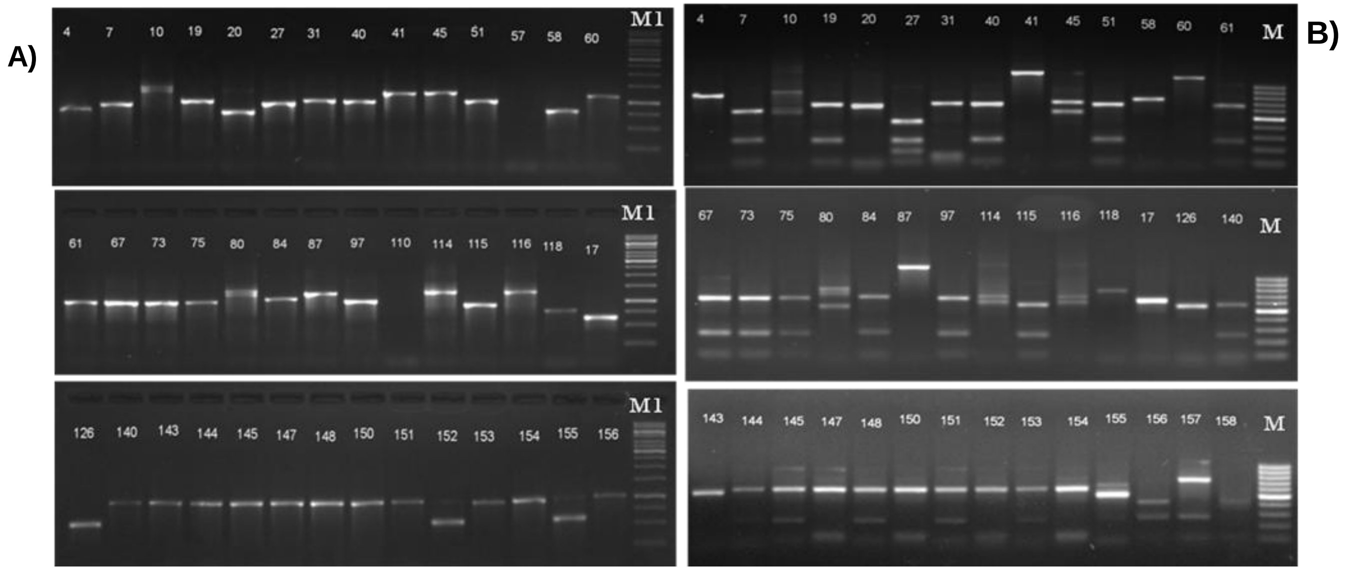

Fig. S1: A) PCR amplified B) *Hae*II digested, IGS (16S-23S rDNA) region of groundnut nodulating isolates;

M1=1kb, M=100bp

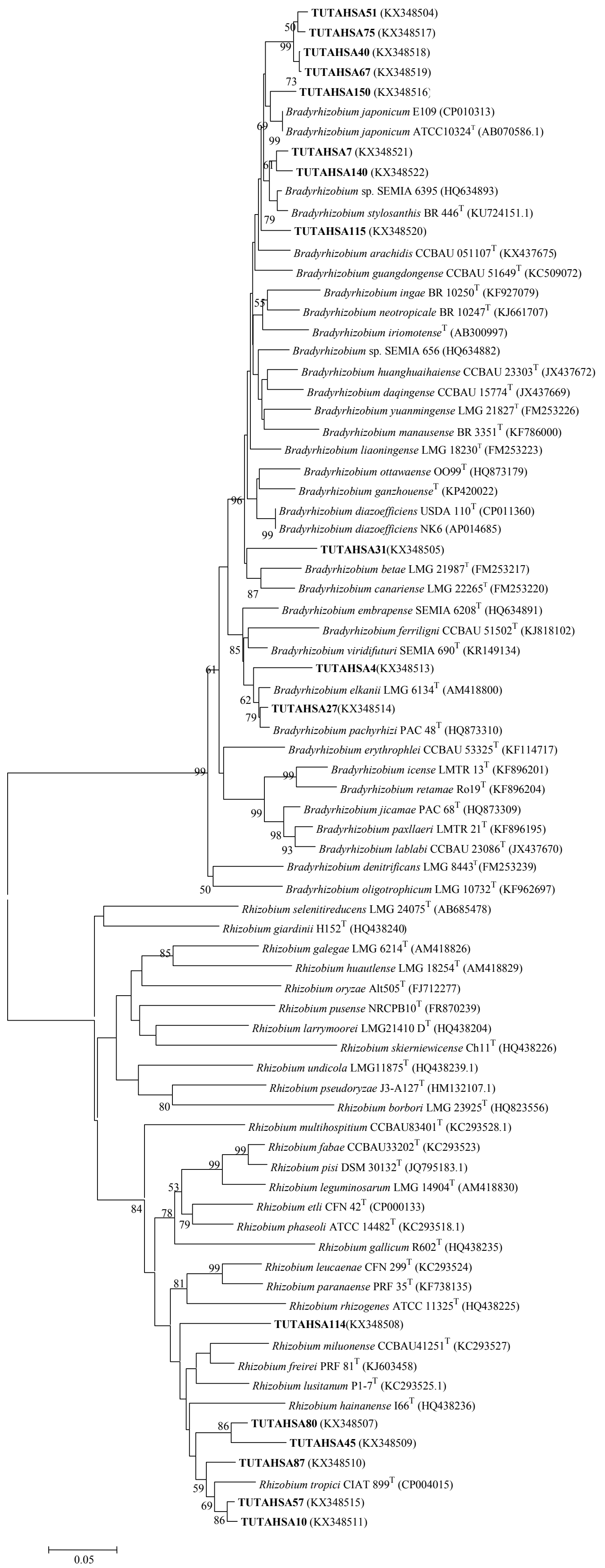

Fig. S2. The neighbour-joining phylogenetic relationships of groundnut nodulating microsymbionts based on *gyrB* sequence analysis. Groundnut nodulating microsymbionts are shown in bold with their nucleotide sequence accession numbers indicated in brackets. The g fi a of a h bra h d at d b a boot trap va u = >50 are indicated for each node (1000 replicates). The scale bar represents the number of changes per nucleotide position. Phylogenetic analyses were conducted in MEGA6.

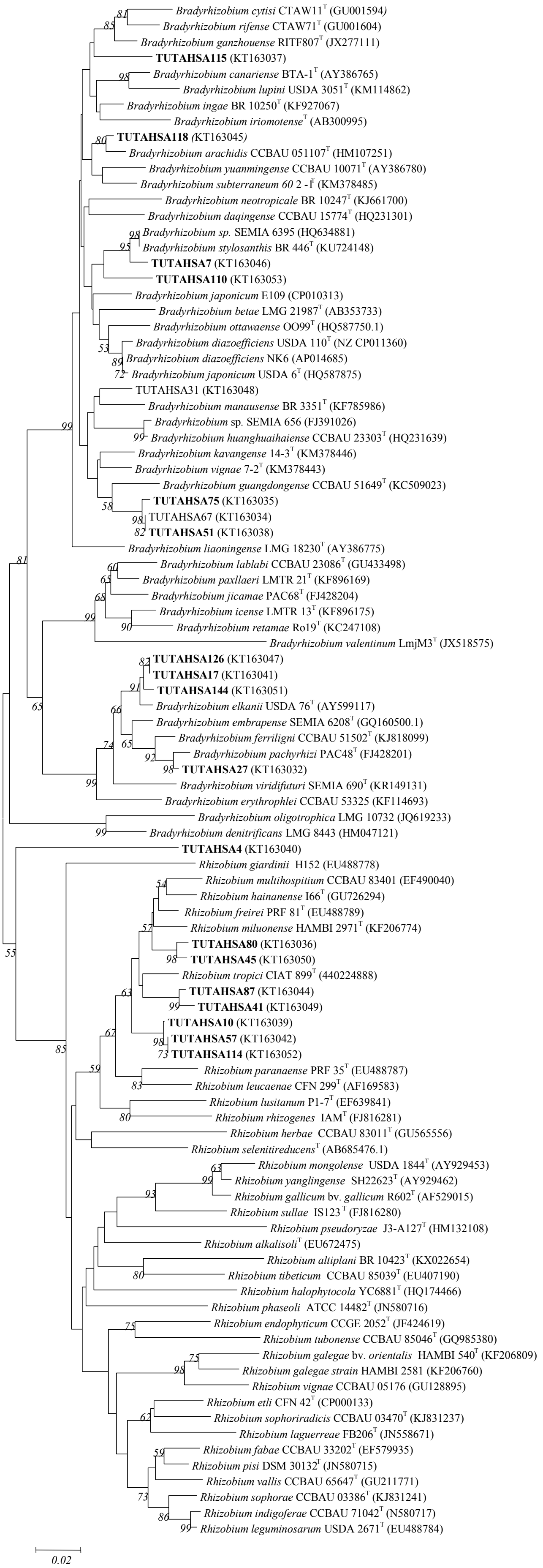

Fig. S3. The neighbour-joining phylogenetic relationships of groundnut nodulating microsymbionts based on *glnII* sequence analysis. Groundnut nodulating microsymbionts are shown in bold with their nucleotide sequence accession numbers indicated in brackets. The significance of each branch is indicated by a bootstrap value = >50 are indicated for each node (1000 replicates). The scale bar represents the number of changes per nucleotide position. Phylogenetic analyses were conducted in MEGA6.

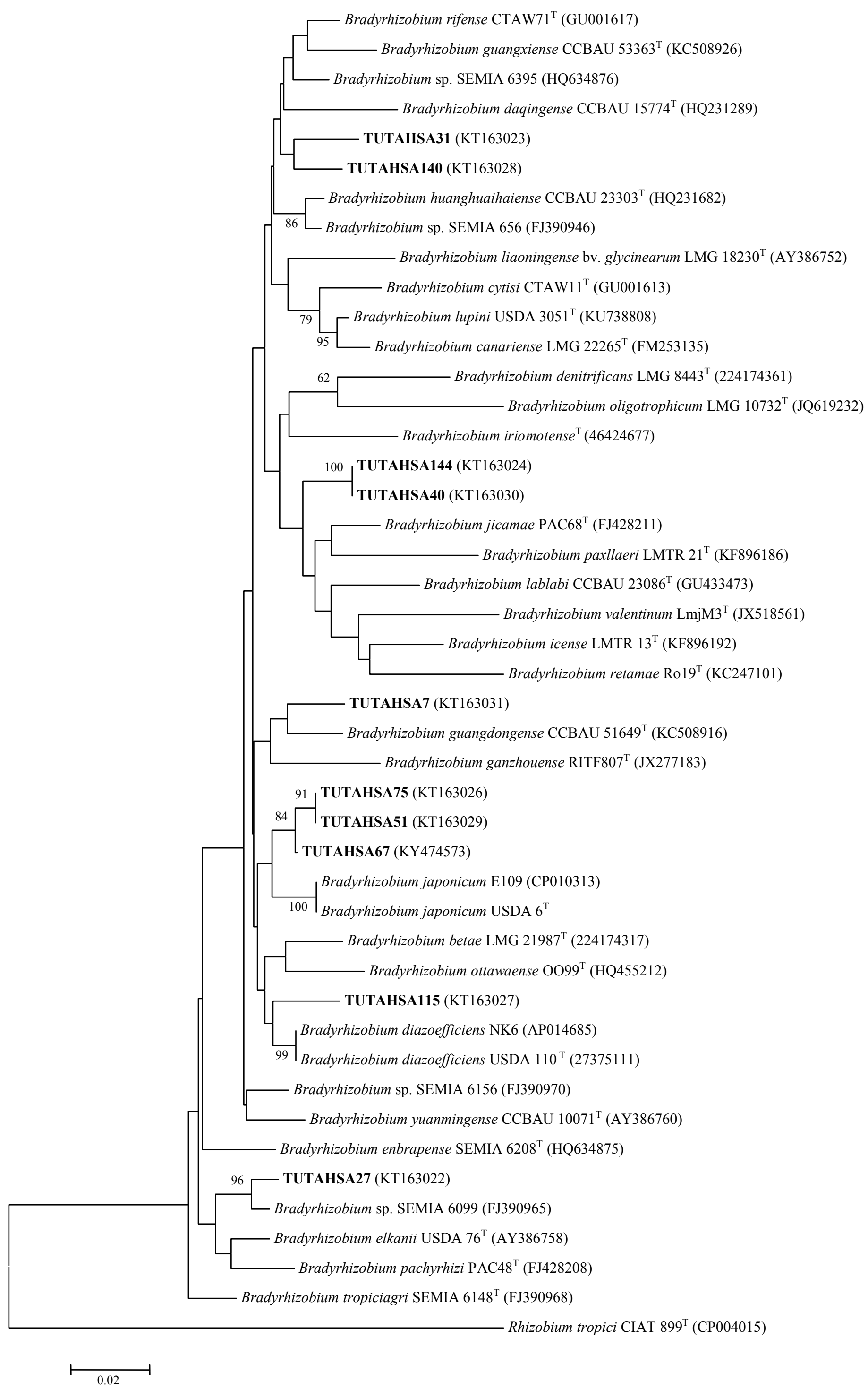

Fig. S4. The neighbour-joining phylogenetic relationships of groundnut nodulating microsymbionts based on *atpD* sequence analysis. Groundnut nodulating microsymbionts are shown in bold with their nucleotide sequence accession numbers indicated in brackets. The significance of each branch is indicated by a bootstrap value = >50 are indicated for each node (1000 replicates). The scale bar represents the number of changes per nucleotide position. Phylogenetic analyses were conducted in MEGA6.

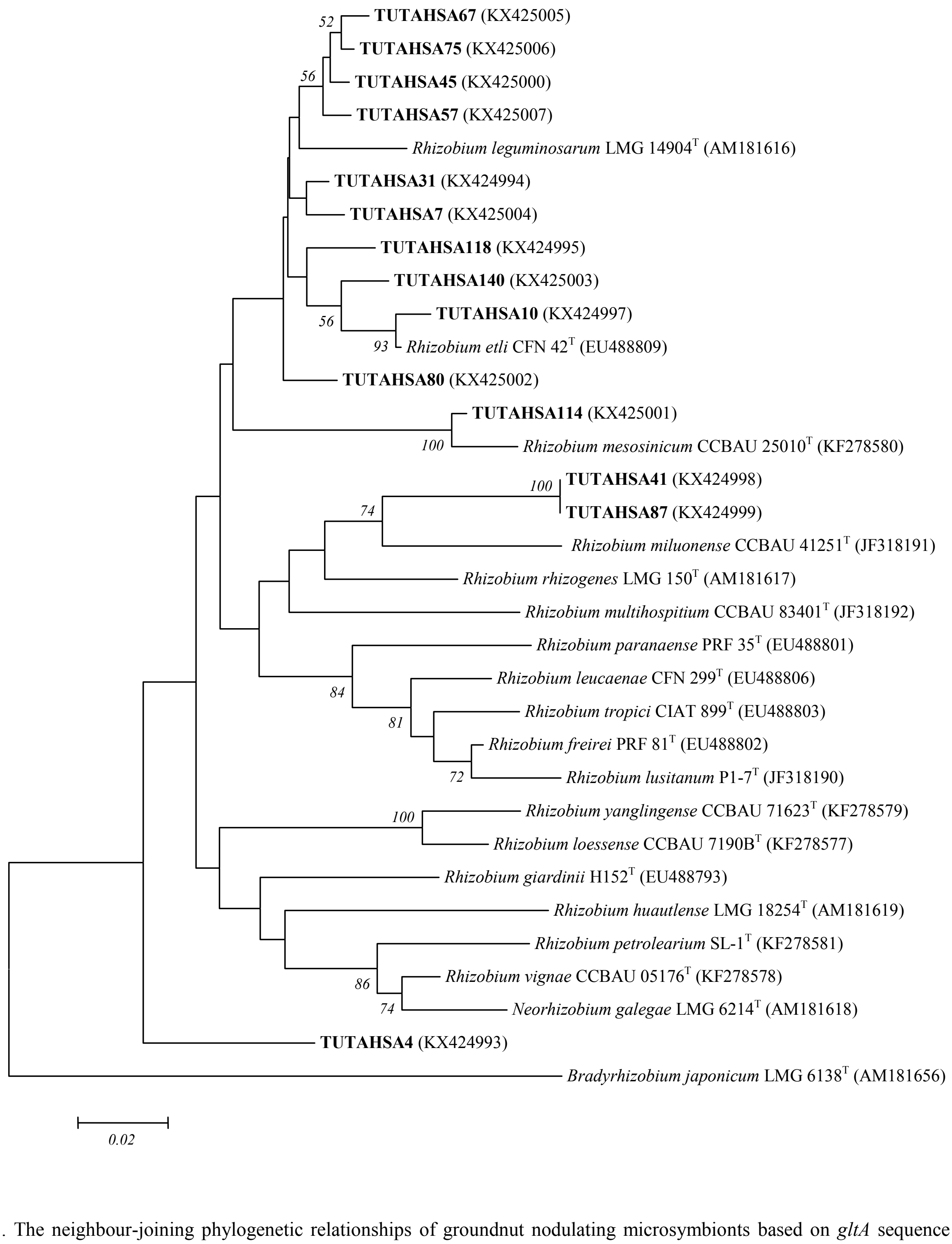

Fig. S5. The neighbour-joining phylogenetic relationships of groundnut nodulating microsymbionts based on *gltA* sequence analysis. Groundnut nodulating microsymbionts are shown in bold with their nucleotide sequence accession numbers indicated in brackets. The g fi a of a h bra h d at d b a boot trap va u = >50 are indicated for each node (1000 replicates). The scale bar represents the number of changes per nucleotide position. Phylogenetic analyses were conducted in MEGA6.
